# Short-Term Azithromycin Treatment Promotes Cornea Allograft Survival in the Rat

**DOI:** 10.1371/journal.pone.0082687

**Published:** 2013-12-09

**Authors:** Katrin Wacker, Sophy Denker, Antonia Hildebrand, Philipp Eberwein, Thomas Reinhard, Johannes Schwartzkopff

**Affiliations:** 1 Eye Center, Albert-Ludwigs-University of Freiburg, Freiburg im Breisgau, Germany; 2 Eye Clinic, Dres Knapp et Schwartzkopff, Lörrach, Germany; Department of Immunology, China

## Abstract

**Background:**

Any inflammatory response following corneal transplantation may induce rejection and irreversible graft failure. The purpose of this study is to analyze the anti-inflammatory effect of azithromycin (AZM) following experimental keratoplasty in rats.

**Methods:**

Corneal transplants were performed between Fisher-donor and Lewis-recipient rats. Recipients were postoperatively treated three times daily with AZM, miglyol, ofloxacin or dexamethasone eye drops. As an additional control, AZM was applied following syngeneic keratoplasty. Furthermore, short-term treatments with AZM for seven days perioperatively or with AZM only three days prior to the transplantation were compared to appropriate controls. All transplants were monitored clinically for opacity, edema, and vascularization. Infiltrating CD45^+^, CD4^+^, CD8^+^, CD25^+^, CD161^+^ and CD163^+^ cells were quantified via immunohistochemistry.

**Results:**

AZM significantly promoted corneal graft survival compared with miglyol or ofloxacin treatment. This effect was comparable to topical dexamethasone. No adverse AZM effect was observed. Histology confirmed a significant reduction of infiltrating leukocytes. The short-term application of AZM for three days prior to transplantation or for seven days perioperatively reduced corneal graft rejection significantly compared with the controls.

**Conclusions:**

Along with antibiotic properties, topical AZM has a strong anti-inflammatory effect. Following keratoplasty, this effect is comparable to topical dexamethasone without the risk of steroid-induced adverse effects. Short-term treatment with AZM three days prior to the transplantation was sufficient to promote graft survival in the rat keratoplasty model. We therefore suggest further assessing the anti-inflammatory function of topical AZM following keratoplasty in humans.

## Introduction

 Corneal transparency is essential for optimal vision. Inflammatory processes at the ocular surface can severely affect corneal transparency because of possible leukocytic infiltration, vascularization and scar formation and subsequent disruption of the ocular immune privilege [1].

 Since the first successful keratoplasty was reported a hundred years ago [2], corneal transplantation has become a standard procedure to restore corneal transparency [3]. Graft survival is excellent, with less than 10% of corneal grafts being rejected [4]. This is mainly due to the immune-privileged status of the eye and the cornea [5] and holds true for low-risk situations, e.g., keratokonus or Fuchs' dystrophy [4]. However, even in these patients, topical corticosteroids are essential as a mainstay treatment during the first months following keratoplasty [6] to reduce corneal inflammation and corneal lymph- and hemangiogenesis [7,8]. 

 In contrast, rejection rates rise comparatively in an infected or vascularized recipient cornea [9,10]. Corneal transplantation in pre-sensitized hosts with surface inflammation, in emergency situations [11], in very young recipients [12,13] or in patients with allergic or atopic afflictions [14,15] shows a higher rate of immune reactions leading to graft failure. Compared to low-risk situations, these patients require systemic immunosuppressive therapy with cyclosporin A or mycofenolate mofetile [10,14] in addition to topical corticosteroids. These systemic treatments are associated with severe adverse effects [6]. Therefore, new formulations and therapeutic strategies to minimize these side effects are currently under investigation [16-18]. 

 Azithromycin (AZM) is a second generation macrolide antibiotic that inhibits mRNA synthesis by binding to the 50S subunit of the bacterial ribosome. As a broad-spectrum antibiotic with a long half-life, AZM is routinely used in the treatment for airway and urogenital infections [19,20]. It has successfully been introduced to treat infectious keratitis, blepharitis and ocular chlamydia trachomatis infections [21-23]. AZM easily penetrates human and rabbit corneas [23,24], even better so in dry eyes [25]. Drug levels remain in a therapeutic range for several days after discontinuation of the application [26,27]. In addition to its antibiotic effect, AZM has been reported to have an anti-inflammatory effect in vitro and in vivo [20,28,29]. Regarding the ocular surface, a reduction of pro-inflammatory cytokines such as IL-1beta and TNF-alpha and infiltrating leukocytes by AZM application has been observed in an experimental model of corneal and conjunctival inflammation [20,30]. Most recently, the beneficial effect of AZM was reported in a mouse model of corneal transplantation [31]. We studied the effects of topically applied AZM in a well-established rat keratoplasty model [12,18] with a particular emphasis on the adequate time frame for treatment. Our data also supports the use of AZM to prevent corneal graft rejection and we strongly suggest that short-term perioperative application is sufficient to abrogate graft failure.

## Materials and Methods

### 1: Animals and groups

 For corneal transplantation, inbred female Fisher (Rt1^lv^) and Lewis (Rt1^l^) rats (Charles River, Sulzfeld, Germany) were used as donors and recipients as previously reported [12,18]. All animals were handled according the EU Directive 2010/63/EU. The protocol was approved by the Committee on the Ethics of Animal Experiments of the Regional Council of Freiburg and the University Medical Center. 

 Eight-week-old Lewis rats were assigned to one of nine groups: Group 1 (n = 6, allogeneic) received dexamethasone eye drops (Monodex, Théa Pharma; Clermont-Ferrand, France) three times daily. Group 2 (n = 16, allogeneic) and group 3 (n = 6, syngeneic) received AZM eye drops (1.5%) (Azytèr, Théa Pharma) three times daily. Group 4 (n = 6, allogeneic) received the azithromycin-solving neutral oil miglyol (Miglyol 812, Caesar& Loretz GmbH, Hilden, Germany). Group 5 (n = 4, allogeneic) received ofloxacin (Floxal EDO, Dr. Mann Pharma GmbH, Berlin, Germany) three times daily. For all groups, therapy was administered for 35 days or until rejection occurred. 

 A short-term treatment regimen of azithromycin for seven days in total (three days prior to transplantation, perioperatively and three days postoperatively) was administered to group 6 (n = 4, allogeneic). Group 7 received miglyol according to the same scheme (n = 4, allogeneic). Groups 8 and 9 (n = 7 each, allogeneic) received azithromycin and miglyol eye drops, respectively, only for three days prior to transplantation (Table 1).

**Table 1:** Groups.

Group	Treatment 3x daily with	End point
1	Dexamethasone	Until rejection occured or day 35
2	Azithromycin	Until rejection occured or day 35
3	Azithromycin, syngeneic	Until rejection occured or day 35
4	Miglyol	Until rejection occured or day 35
5	Ofloxacin	Until rejection occured or day 35
6	Azithromycin	Seven days, starting three days prior to keratoplasty
7	Miglyol	Seven days, starting three days prior to keratoplasty
8	Azithromycin	Three days prior to keratoplasty
9	Miglyol	Three days prior to keratoplasty

### 2: Corneal transplantation and anesthesia

 Anesthesia was introduced by isoflurane (ABBOTT GmbH&Co.KG, Wiesbaden, Germany) inhalation and maintained by the intraperitoneal application of xylazine (Bayer, Leverkusen, Germany), ketamine (Essex, München, Germany) and atropine (Braun, Melsungen, Germany). Orthotopic penetrating keratoplasties were performed as previously described [12,18]. In brief, the rats were anesthetized as described above. Fisher donor buttons (2.5 mm) were obtained and the animals were euthanized afterwards. The central corneas of the Lewis recipients were trephined (2.0 mm) and the donor corneas were fixed with eight interrupted sutures (11.0 Ethilon, Ethicon, Norderstedt, Germany). Following keratoplasty, a blepharorrhaphy was applied for one day to protect the graft. 

### 3: Clinical graft assessment

 Two independent investigators blinded to treatment groups performed the clinical examination. Signs of opacity, vascularization and corneal edema were evaluated as described previously [18] (Table 2). Opacification of the graft was scored as follows: 0 = no opacity; 1 = slight opacity, details of iris clearly visible; 2 = moderate opacity, some details of iris not visible; 3 = strong opacity, pupil still recognizable; and 4 = total opacity. Rejection was defined as complete graft opacification (grade 4). Vascularization was scored as follows: 0 = no vessels; 1 = vessels on host; 2 = vessels in the periphery of the transplant; and 3 = vessels reaching the center of the transplant. Edema was scored as follows: 0 = no edema; 1 = slight edema; 2 = strong edema, margin of the transplant slightly elevated; and 3 = severe edema, margin of the transplant severely elevated [12].

**Table 2:** Clinical Graft Assessment Score.

Opacification	
0	No opacity
1	Slight opacity, details of the iris clearly visible
2	Moderate opacity, some details of the iris no longer visible
3	Strong opacity, pupil still recognizable
4	Total opacity, pupil no longer visible
Vascularization	
0	No vessels
1	Vessels on host not in the transplant
2	Vessels in the periphery of the transplant
3	Vessels reaching the center of the transplant
Edema	
0	No edema
1	Slight edema
2	Strong edema, transplant margin slightly elevated
3	Severe edema, transplant margin elevated

### 4: Histological analyses of the corneal infiltrate

 On day 13 following keratoplasty, the rats were euthanized to conduct a immunohistological evaluation. CD45^+^ leukocytes, CD4^+^ T cells, CD8^+^ T cells, CD25^+^ T cells, CD161^+^ NK cells and CD163^+^ macrophages were stained on cryosections as described previously [12]. Snap-frozen eyes were sliced in 6-µm cryosections and fixed in acetone at –20°C. After blocking unspecific bindings with TRIS buffers containing 10% of calf serum, mouse anti-rat antibodies (anti-CD45: clone IBL-3/16; anti-CD4: clone W3/25; anti-CD8: clone OX-8; anti-CD25: clone OX-39; anti-CD163: clone ED2; and anti-CD161: clone 10/87—all from AbD Serotec, Düsseldorf, Germany) were applied to the sections. Subsequently, a biotinylated rabbit anti-mouse secondary antibody (Dako, Hamburg, Germany) was incubated and followed by streptavidin-conjugated alkaline phosphatase (Dako, Hamburg, Germany). In the following step, the corresponding substrate (Vector, Burlingame, VT, USA) was incubated and the sections were counterstained with Mayer’s hematoxylin. Two independent and blinded investigators counted positively stained cells within three squares in the central corneal stroma, and the mean cellular infiltrate per mm^2^ was calculated. Stained slices of rat spleen served as positive and negative controls.

### 5: Statistics

 Rejection was analyzed using the Kaplan–Meier estimator and compared with the log-rank test between the groups. The densities of infiltrating immune cells in corneal allografts were compared with two-tailed student's t-tests. An alpha level of 5% was considered statistically significant.

## Results

### 1: Topical AZM improves corneal graft survival

**Figure 1:**
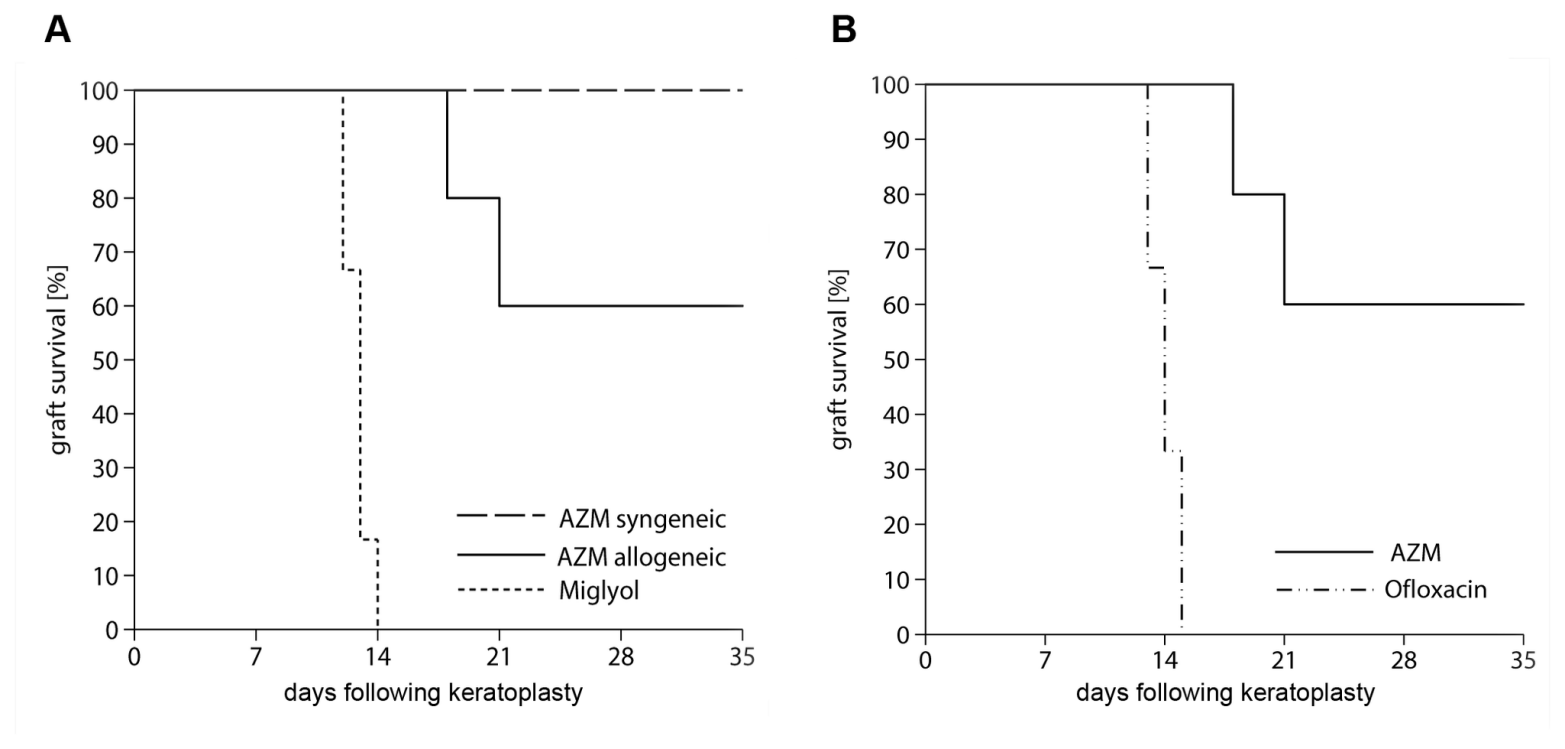
Graft survival. (A) Clear graft survival following keratoplasty is shown by Kaplan-Meier estimator. Treatment with miglyol (group 4) led to rejection at day 13 [12.5–13.5], whereas with AZM (group 3), no rejection was detected. When treating the allogeneic transplanted animals with AZM (group 2), rejection occurred at day 28.5 [20.3–36.6] postoperatively. AZM allogeneic compared with the control with miglyol did promote graft survival (p < 0.004). (B) Treatment with ofloxacin (group 5) led to rejection at day 13, similar to miglyol, while AZM improved graft survival significantly.

 No rejection was observed in the syngeneic group treated with AZM. All allogeneic grafts treated with miglyol (group 4) were rejected around postoperative day 13. In contrast, a significant number of corneal transplants (> 65%) in recipients that received topical AZM (group 2) survived throughout the observation period (p < 0.01 compared to miglyol). No beneficial effect of the topical ofloxacin treatment was seen, with 100% rejection occurring in group 5 (Figure 1).

### 2: Comparison of topical AZM and topical corticosteroid treatment

**Figure 2:**
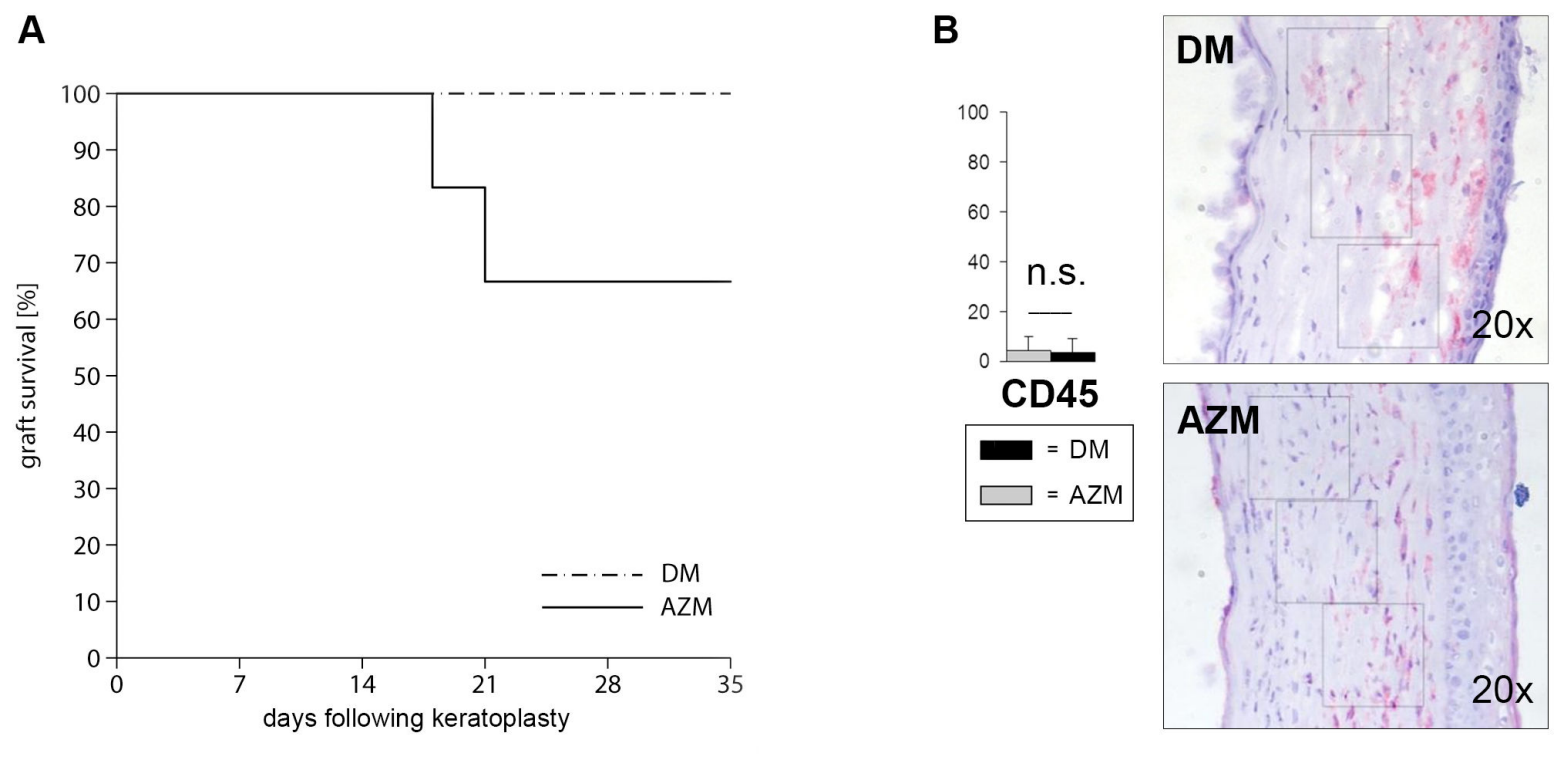
Clinical graft evaluation of azithromycin and dexamethasone following keratoplasty. (A) Orthotopic penetrating keratoplasties between Lewis and Fisher rats were performed. Total graft survival post keratoplasty is shown by Kaplan-Meier curves. Rejection was defined as total graft opacification (opacity grade 4). With AZM (group 2), rejection occurred at day 28.5 [20.3–36.6] postoperatively. No rejection occurred in the DM-treated group (group 1) (p < 0.14). (B) Immunostainings on cryosections of DM-treated (control, black) and AZM-treated animals (light grey). Total leukocytic infiltrate was counted in three different squares per animal by two independent investigators. No significant difference of total leukocytic infiltrate (red cells) between the group 1 and 2 could be detected at day 35. Bars show group 1 (control, black) in comparison to group 2 (grey) with SEM.

 No rejection occurred at any time point if topical dexamethasone was applied. This effect was clinically superior to the AZM treatment, in which some grafts were rejected during the observation period (Figure 2a). To analyze differences in the inflammatory responses in the graft in more detail, infiltrating CD45+ leukocytes were stained histologically in the graft at postoperative day 35. No significant differences in the amount of infiltrating cells were found (Figure 2b). 

### 3: Topical AZM treatment reduces clinical signs of inflammation after keratoplasty

**Figure 3:**
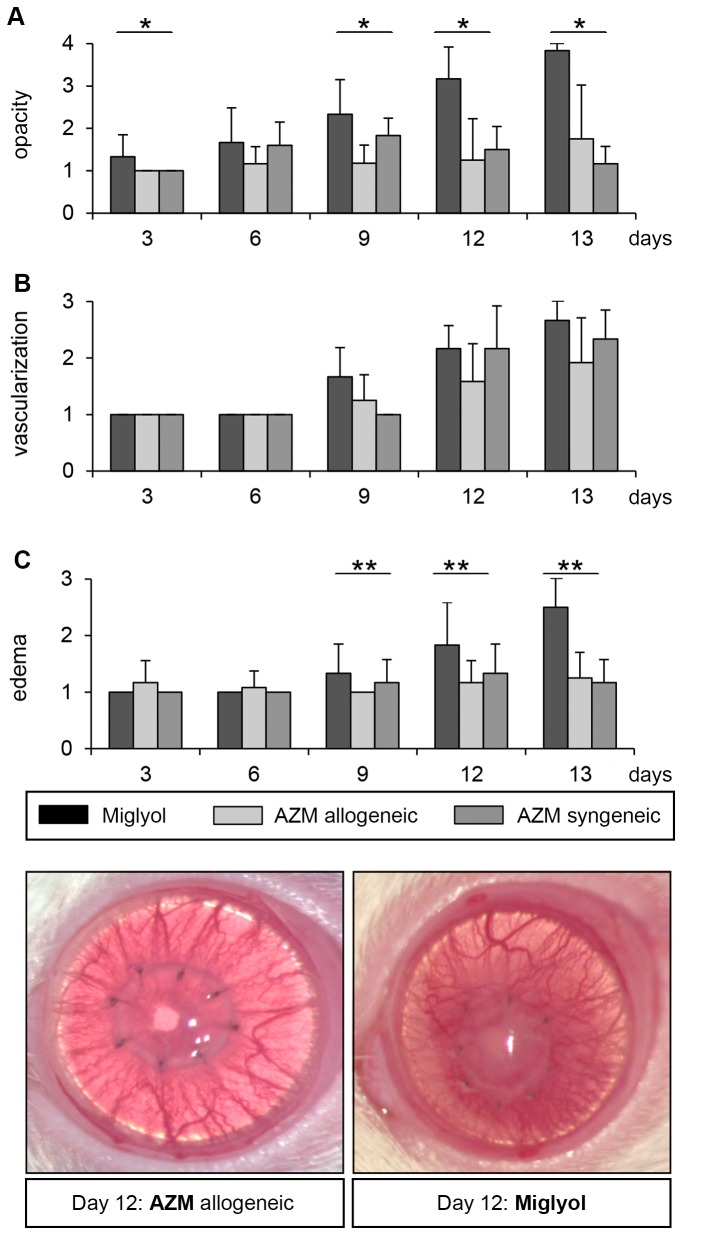
Clinical graft evaluation of AZM compared to miglyol. (A) Opacification, vascularization, and edema were monitored every three days by two independent investigators. A significant difference in opacification between the miglyol and AZM allogen group could be detected at day 3 (p < 0.05), day 9 (p < 0.01) and at day 12 (p < 0.001) after keratoplasty. No significant difference between AZM syngeneic (group 3) and allogeneic (group 2) could be detected. Vascularization was not influenced in all groups. (B) Vascularization was not affected by AZM compared to controls. (C) Corneal edema was reduced in the AZM allogen group compared to the miglyol group beginning at day 9 (p < 0.05). A representative photo on day 12 following keratoplasty is shown. Bars show group 4 (control, black) in comparison with group 2 (grey) and group 3 (control, light grey) with SEM.

 Grafts that had been treated with topical miglyol (group 4, solvent control) were rejected on postoperative day 13 (Figure 1). All clinical signs of inflammation increased to a peak on day 13 (Figure 3). Syngeneic corneal grafts treated with AZM did not develop a comparable opacification or edema. In contrast, in the allogeneic group 2, which had been treated with topical AZM, scores for opacification and edema formation were significantly lower compared to the miglyol controls (p < 0.05). No effect on corneal vascularization was detected in any of the groups.

### 4: Influence of topical AZM on leukocytic infiltration following corneal transplantation

**Figure 4:**
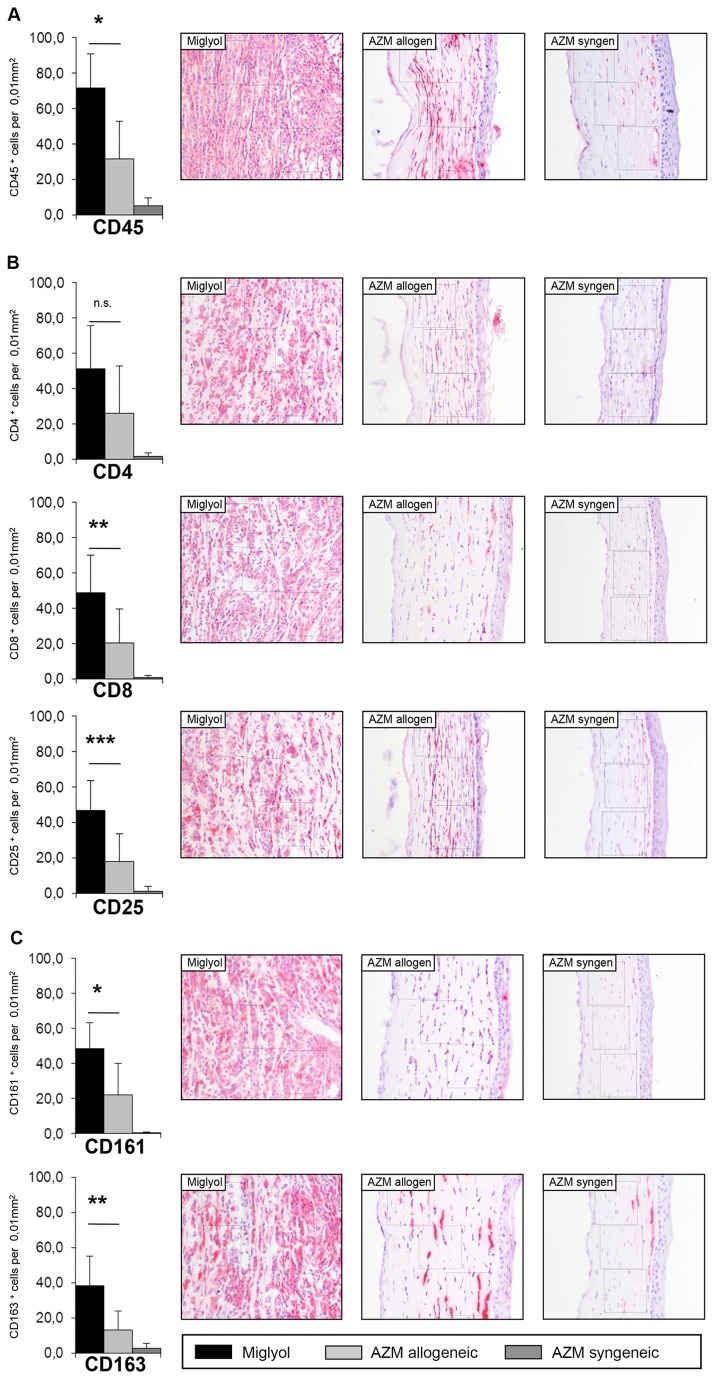
Immunohistological analysis of leukocytic infiltration. (A) Immunostainings on cryosections of miglyol-treated (group 4, black) and AZM-treated animals (group 2, light grey; group 3, dark grey) were performed on day 13. Infiltrating CD45^+^ leukocytes in group 2 were significantly reduced in comparison to group 4 (p<0.01). Red cells are positive for the antibody. (B) While no difference in the number of CD4^+^ T cells was seen in both groups, the infiltrating CD8^+^ T cells (p < 0.05) and CD25^+^ T cells (p < 0.05) were reduced significantly in the AZM-treated animals. (C) The number of infiltrating CD161^+^ NK cells (p < 0.05) and of CD163^+^ macrophages (p < 0.05) in the central corneal stroma was significant reduced.

 Regarding the total leukocyte infiltration, a significant reduction was achieved by AZM compared to miglyol treatment (p < 0.01) (Figure 4a). The infiltrate of CD4^+^, CD8^+^ and CD25^+^ cells was reduced in the AZM-treated grafts (p < 0.05 for CD8^+^ and CD25^+^; Figure 4b). The amount of CD161^+^ NK cells and CD163^+^ macrophages was also significantly lower following AZM treatment (p<0.05; Figure 4c). Only a minor cellular infiltrate was observed in the syngeneic grafts. Generally, graft edema was also decreased in the histological sections in the allogeneic AZM group compared with the allogeneic miglyol group.

### 5: Short-term topical AZM is sufficient to promote corneal graft survival

**Figure 5:**
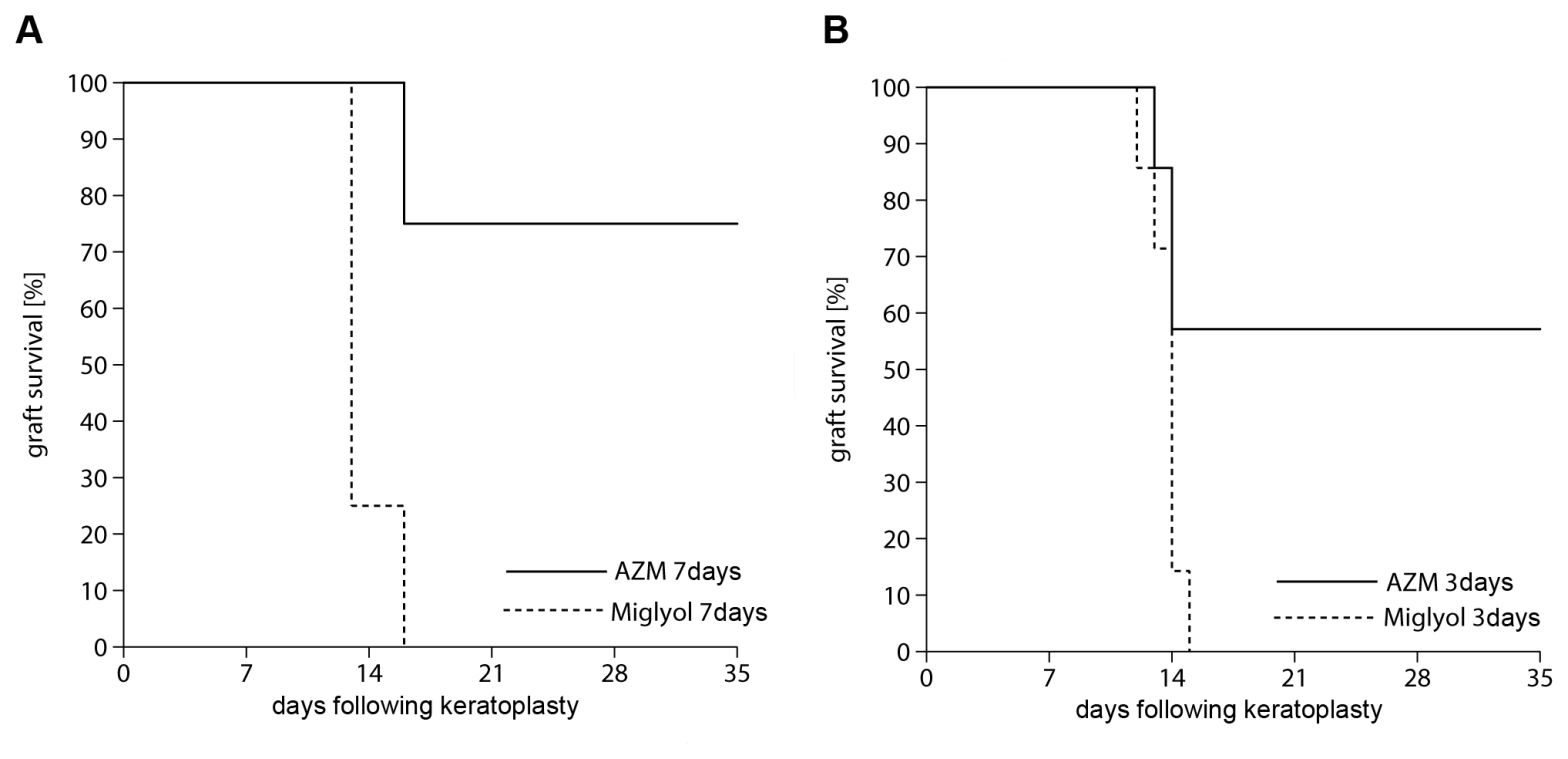
Short-time treatment with AZM. (A) A reduced treatment regimen with seven days in total (three days prior to transplantation and three days following keratoplasty) in group 6 improved graft survival significantly compared to controls in group 7 (p < 0.05). Rejection occurred in miglyol-treated rats at day 14 [12.3–15.2], whereas in the AZM-treated group, rejection occurred at day 30 [22.2–38.1]. (B) Even only a three-day preoperative treatment with AZM (group 8) improved graft survival significantly compared with controls with miglyol (group 9; p < 0.05). Rejection occurred in group 9 at day 13 [13.0–14.4], whereas in group 8, rejection occurred at day 26 [18. –33.7].

 The reduced seven-day treatment with AZM (group 6) showed significantly improved allograft survival compared with group 7 (migylol; p < 0.05; Figure 5a). In the miglyol-treated group, rejection occurred at day 13. Three-day AZM treatment prior to keratoplasty (group 8) was sufficient to significantly increase corneal graft survival compared with the control group 9. (p < 0.05; Figure 5b).

## Discussion

 Corneal transplantation is the standard procedure to restore corneal transparency and improve vision. An optimal postoperative treatment should not only control the inflammatory response efficiently and be applied topically, but also be kept as short as possible to reduce adverse effects. 

 Azithromycin (AZM) is a well-known antibiotic agent approved for topical use against ocular surface infections [21–26]. AZM acts as an anti-inflammatory agent modifying the production of inflammatory cytokines that play an important role in corneal graft rejection [20,28–30]. Therefore, we hypothesized that 1.5% AZM drops would affect the inflammatory process following keratoplasty and thereby prolong graft survival.

 Topical AZM prolonged graft survival significantly in the rat keratoplasty model compared with its solvent miglyol (p<0.01; Figure 1a). To exclude side effects, syngeneic grafts were similarly treated topically with AZM. No clinically obvious inflammation was observed (Figure 1a). Therefore, we excluded a negative effect of AZM on corneal transplants. 

 In addition to its anti-inflammatory effect, AZM is bactericidal. The bacterial flora of the ocular surface is affected by antibiotic treatment, possibly changing the immune response triggered by the corneal graft. To address the possible influence of a reduced bacterial load on allograft survival, we treated allogenically transplanted rats with non-conserved antibiotic ofloxacin in an additional control group. All ofloxacin-treated grafts were rejected. No significant difference to the animals treated with miglyol was detected (Figure 1a). Despite the anti-inflammatory benefit of topical AZM, its continuous application should be evaluated critically considering that antibiotics could favor the selection of antibiotic-resistant bacteria. 

 We also compared the anti-inflammatory effect of AZM with the effects of the current first-line therapy with topical corticosteroids. Dexamethasone (DM) is a very effective anti-inflammatory agent but associated with a plethora of short-term and long-term adverse effects, e.g., the feared steroid response that jeopardizes the transplant and the vision itself. All grafts treated with DM survived. Topical treatment with DM was superior to treatment with AZM (Figure 1a). Histologically, a minimal corneal infiltrate in both AZM- and DM-treated grafts (groups 1–3) could be detected (Figure 2). From these results, we concluded that DM, the mainstay treatment following keratoplasty, is superior and should still be administered as a first-line treatment. However, AZM might be a valuable additive to reduce steroid use and associated side effects (IOP elevation, cataracts). 

 To further analyze the anti-inflammatory effect of AZM on inflammation following keratoplasty, we examined the inflammation clinically and histologically. Clinically, the inflammation was quantified by a score of corneal opacification, edema-formation and corneal vascularization. This score followed internationally accepted standards [12,18]. As demonstrated in Figure 3, opacity and edema were significantly lower in the AZM-treated group compared with the miglyol group. No effect was seen with respect to blood vessel formation. This is consistent with the results obtained by Sadrai et al. [30], who found that corneal neovascularization induced by VEGF A pellets was not affected by AZM in a mouse model. In our model, this may be explained by the fact that not only the graft but also the remaining sutures induced corneal vascularization. This would be consistent with the observation that a similar amount of vessels is formed following syngeneic transplantation. Irrespective of the tissue studied or animal model chosen, AZM does not seem to have an effect on neovascularization. 

 Our clinical results were further confirmed by histological analyses. The total leukocytic infiltration was significantly reduced by AZM. This applied to all cell types studied (Figure 4 a-c.) Despite a reduction of all these cell types, no significant difference was observed for total CD4^+^ T cells. This finding was especially interesting given that CD4^+^ T cells are known to be the critical element that leads to corneal graft rejection [32,34]. However, several studies indicated that aside from CD4^+^ T cells, different elements of the immune system mediate graft rejection following keratoplasty [9,33]. We therefore suggest that AZM has a general anti-inflammatory effect that is weakest with respect to CD4^+^ T cell inhibition. However, this remains to be determined in further in vitro analyses.

AZM is used in pulmonology, based on the observation that it reduces the levels of inflammatory cytokines such as IL-1beta and TNF-alpha in the lung [20,29,30]. An IL-10-mediated effect on dendritic cells in vitro is also reported [28]. These cells and cytokines are known to play an important role during corneal graft rejection [33]. This might explain why the effect of AZM on CD4^+^ T cells becomes negligible and AZM has a statistically significant effect on graft survival in the rat keratoplasty model. 

 Our results are in line with very recent observations reported by Medina et al. [31] that demonstrated that AZM promotes corneal graft survival in a mouse model for high-risk keratoplasty. Similarly, we did not observe altered T-cell responses in draining lymph nodes of AZM-treated recipients with respect to cytokine production or proliferative capacity (data not shown). In addition, we provided evidence to suggest that topical application of AZM is sufficient to reduce corneal inflammation and that the treatment period can be reduced to three days prior to transplantation without losing a graft-survival promoting effect. This is especially important given the risk of emerging bacterial resistances. We excluded an effect by alteration of the bacterial flora using appropriate controls.

 In summary, we confirm in the rat keratoplasty model that topical AZM has sufficient anti-inflammatory capacity to prevent corneal graft rejection. In addition, we showed that three days of topical preoperative treatment suffice to improve graft survival. The Food and Drug Administration has approved AZM (AZASite, Inspire Pharmaceuticals Inc., Durham, USA) as an antibiotic with a treatment duration of seven days and the administration of Azyter for three days. Even though the exact mechanism of action of AZM in transplant preservation remains incompletely understood, we suggest investigations of the topical application of AZM following corneal transplantation in clinical trials. The standard treatment scheme being a combination of steroids and antibiotics, AZM would both fulfill anti-bacterial and anti-inflammatory functions. The advantages of AZM are its topical applicability and the already existing approval. Its advantageous side effect profile as topical antibiotic was confirmed after keratoplasty in a small single center study in humans [35]. Our promising results for AZM following keratoplasty support clinical studies in humans of AZM as an anti-inflammatory agent with additive antibiotic function that acts together with topical steroids.
