# Correction: Short-Term Azithromycin Treatment Promotes Cornea Allograft Survival in the Rat

**DOI:** 10.1371/journal.pone.0102639

**Published:** 2014-07-07

**Authors:** The PLOS ONE Staff

## Notice of Republication

This article was republished on June 18, 2014, due to the figures missing in the PDF version of the previously published article. The originally published, uncorrected article and the republished, corrected article are provided here for reference.

## Supporting Information

File S1 Originally published, uncorrected article.
(PDF)

File S2 Republished, corrected article.
(PDF)
